# Surgical Site Infection in Microvascular Free Flap Reconstruction of the Head and Neck: An Analysis of Risk Factors

**DOI:** 10.1111/ans.70512

**Published:** 2026-02-12

**Authors:** Marco Stocca, Pascalino Romeo, Yasiru Gehan Karunaratne, Bishoy Soliman, Thomas C. Lam, Huang‐Kai Kao, Frank Hsieh

**Affiliations:** ^1^ Sydney Medical School Sydney University New South Wales Australia; ^2^ Department of Plastic and Reconstructive Surgery Westmead Hospital Sydney Australia; ^3^ Faculty of Medicine and Health Sciences Macquarie University Sydney Australia; ^4^ Department of Plastic and Reconstructive Surgery Chang Gung Memorial Hospital & Chang Gung University College of Medicine Taoyuan Taiwan

**Keywords:** head and neck free flap, head and neck reconstruction, prophylactic antibiotics, surgical site infection

## Abstract

**Backgrounds:**

Free flap reconstruction has become the standard of care in the repair of complex defects of the head and neck; however, surgical site infection remains a key area for improvement. This study aims to better understand the risk factors for surgical site infection in head and neck free flap reconstruction and gain insight into antibiotic prophylaxis choices.

**Methods:**

This retrospective cohort study reviews data from 100 cases across a 5‐year period. Univariate and multivariate analyses have been employed to elucidate statistically significant associations, examining surgical site infection, potential risk factors and other post‐operative markers.

**Results:**

A surgical site infection developed in 32.0% of cases, with an average onset of 11.3 days post‐operation. Preoperative anaemia (OR: 3.259) and a prophylactic antibiotic duration of ≤ 24 h (OR: 3.010) were found to be independently significant risk factors for infection. Furthermore, surgical site infection was found to significantly increase unplanned returns to theatre (*p* ≤ 0.001), complete flap failure (*p* = 0.005) and length of stay (*p* = 0.026). The results also suggest that a 48‐h prophylaxis duration may be optimal, with no significant difference in infection rate at this cut‐off point.

**Conclusion:**

To the author's knowledge, this is the first paper to establish preoperative anaemia as an independently significant risk factor for surgical site infection in head and neck free flap operations. This study also finds the Australian guidelines' recommendation of ≤ 24 h of antibiotic prophylaxis to be potentially inadequate in effectively controlling surgical site infection following head and neck free flap reconstruction.

## 1 Introduction

Microvascular reconstruction is the gold standard in the treatment of complex defects in the head and neck. Free flaps are a reliable and sophisticated reconstructive option enabling surgeons to reduce morbidity associated with head and neck cancer while improving functional, oncological and aesthetic outcomes [1]. The incidence of major complications in microsurgery has reduced as techniques have been refined [2]. In the head and neck, however, surgical site infection (SSI) continues to be a significant source of morbidity [3, 4, 5, 6]. The anatomy and extent of many of these procedures, coupled with the long duration of operation, can predispose to SSI if care is not taken.

Considering the unfavourable consequences associated with infection, guidelines should be up to date to effectively reduce the incidence of this complication. Recent studies, however, have reported rates of SSI following head and neck free flap reconstruction ranging from less than 10% to over 40% in some major hospitals [5, 7, 8, 9, 10, 11, 12, 13, 14]. These figures suggest current practices and recommendations may benefit from review and optimisation. Current Australian guidelines recommend cefazolin monotherapy or a cefazolin‐metronidazole combination as the first‐line prophylaxis options, with clindamycin recommended in penicillin‐allergic patients for a period of no longer than 24 h [15].

This study aims to better understand risk factors for SSI in microvascular free flap reconstruction of the head and neck in an Australian context. We also aim to gain greater clarity and insight into the effects of antibiotic duration on SSI in these complex operations.

## 2 Methods

This study was approved by the Western Sydney Local Health District Research Governance Office (ETH00868). Data were collected from the electronic medical records of adult patients who underwent head and neck surgery with free flap reconstruction at Westmead Hospital from January 2018 to May 2023. A total of 100 patients were included, 71 male and 29 female, with a mean age of 62.3 years at the time of surgery (range: 31.5–87.2). The study period was defined pragmatically to capture a contemporary dataset reflective of current practice; the resulting cohort of 100 patients was coincidental rather than a predetermined sample size. Sixty percent of the cohort had smoked at some point during their lives, with 29.0% being active smokers. A quarter of the participants consumed 10 or more standard drinks per week and were categorised as heavy alcohol users. Prior radiotherapy to the surgical field had been performed in 14.0% of the cohort, and 24.0% were diagnosed with diabetes mellitus. The majority (*n* = 51) of participants were assessed to have an ASA score of 3 before surgery, 39 participants had a score of 2, 8 participants received a score of 4 and the remaining 2 participants received a score of 1. Following the World Health Organisation definition, participants were determined to have preoperative anaemia if haemoglobin concentration was < 130 g/L in males and < 120 g/L in females [16]. Preoperative hypoalbuminemia was defined as an albumin concentration of < 35 g/L [17]. From those with data available, only 8.4% had hypoalbuminemia before surgery, whilst almost a quarter (24.2%) were found to be anaemic.

The indication for surgery in 94.0% of cases was cancer, with the remaining split between flap revisions (*n* = 3), osteoradionecrosis (*n* = 2) and commissuroplasty (*n* = 1). 67.0% of the operations involved disruption of the alimentary tract or trachea, with the surgical site considered to be ‘clean‐contaminated’ for 73.0% of the cohort and ‘clean’ for the remaining 27.0%. A tracheostomy was performed in 69.0% of cases and 86.0% involved a neck dissection. 76.7% of neck dissections were unilateral, with the remaining 23.3% being bilateral.

The primary outcome for this study is SSI occurring within 30 days of the procedure. SSI was defined according to specific *Centers for Disease Control and Prevention* (CDC) criteria for superficial and deep incisional SSI, as is consistent with the current literature, and was assessed at the recipient site [18].

### 2.1 Statistical Analysis

Statistical analyses on this data were performed using Jamovi (version 2.3.26) [19]. The chi‐squared test was applied for categorical data, while the Student's *t*‐test was utilised for continuous data. The Mann–Whitney *U* test was employed for one continuous variable where Levene's test was significant (< 0.05), suggesting a violation of the assumption of equal variances. These tests were applied to determine significant relationships between the potential risk factors and our primary outcome. Risk factors determined to have a significant relationship on univariate analysis underwent multivariate analysis (binomial logistic regression) to assess independent significance. Our primary outcome also underwent univariate analyses against our secondary outcomes to determine any significant relationship between SSI and other post‐operative markers. A *p* < 0.05 was deemed significant.

## 3 Results

Thirty‐two percent of patients developed an SSI, with an average onset of 11.3 days post‐operation (range: 2–28 days). The median length of stay for the cohort was 14 days (interquartile range = 12.5 days), with 32.0% of patients requiring an unplanned return to theatre. Flap success was within acceptable ranges, with complete flap failure occurring in 6 cases and partial flap failure occurring in 13 cases. On a univariate analysis of the secondary effects of SSI (Table 1), SSI was found to be significantly related to unplanned return to theatre (*p* < 0.001), complete flap failure (*p* = 0.005) and length of stay (*p* = 0.026). SSI was not found to be a significant risk factor for partial flap failure (*p* = 0.070). Regarding risk factors for SSI, preoperative anaemia (*p* = 0.002) and an antibiotic prophylaxis duration of ≤ 24 h (*p* = 0.013) were both found to significantly increase SSI rates on univariate analysis (Table 2). The duration of antibiotic prophylaxis was also dichotomised into ≤ 48 and > 48 h sub‐groups; however, unlike the 24‐h cut‐off, there was no significant difference in SSI rate (*p* = 0.311). A complete outline of the univariate analyses can be found in Table 2. The variables found to be significant on univariate analysis were then included in a binomial logistic regression analysis to test for independent significance (Table 2). Preoperative anaemia (*p* = 0.025, OR: 3.259; 95% CI: 1.159–9.169) and an antibiotic prophylaxis duration of ≤ 24 h (*p* = 0.041, OR: 3.010; 95% CI: 1.045–8.674) were found to remain significant on a multivariate analysis.

**TABLE 1:** Post‐operative complications.

Variable	SSI, *n* = 32 (32.0%)	No SSI, *n* = 68 (68.0%)	Total (*N* = 100)	*p*
Length of stay (days); mean (SD)	28.2 (34.2)	16.9 (15.9)	20.5 (23.8)	**0.026**
Unplanned return to theatre				**< 0.001**
Yes	18 (56.3%)	14 (20.6%)	32 (32.0%)	
No	14 (43.8%)	54 (79.4%)	68 (68.0%)	
Partial flap failure				0.070
Yes	7 (21.9%)	6 (8.80%)	13 (13.0%)	
No	25 (78.1%)	62 (91.2%)	87 (87.0%)	
Complete flap failure				**0.005**
Yes	5 (15.6%)	1 (1.5%)	6 (6.00%)	
No	27 (84.4%)	67 (98.5%)	94 (94.0%)	

*Note*: Bold values are statistically significant.

**TABLE 2:** Statistical analysis of risk factors for SSI.

Univariate analysis				
Variable	SSI, *n* = 32 (32.0%)	No SSI, *n* = 68 (68.0%)	Total (*N* = 100)	*p*
Continuous variables; mean (SD)				
Age (years)	64.4 (11.1)	61.3 (13.2)	62.3 (12.6)	0.254
BMI (kg/m^2^)	28.3 (7.30)	27.8 (5.87)	28.0 (6.33)	0.714
Albumin (g/L)	38.5 (5.28)	40.2 (3.57)	39.6 (4.34)	0.083
Haemoglobin (g/L)	130 (20.4)	137 (15.2)	135 (17.3)	0.075^a^
Surgical duration (h)	11.7 (3.49)	11.6 (2.96)	11.7 (3.12)	0.981
Ischaemic duration (min)	108 (62.0)	111 (43.7)	110 (50.2)	0.821
Antibiotic duration (days)	3.38 (3.40)	4.03 (3.32)	3.82 (3.34)	0.363
Categorical variables; number (%)				
Sex				0.416
Male	21 (65.6%)	50 (73.5%)	71 (71.0%)	
Female	11 (34.4%)	18 (26.5%)	29 (29.0%)	
BMI				0.590
Healthy weight^b^	10 (31.3%)	25 (36.8%)	35 (35.0%)	
Unhealthy weight^c^	22 (68.8%)	43 (63.2%)	65 (65.0%)	
Smoking history				0.991
Current smoker	9 (28.1%)	20 (29.4%)	29 (29.0%)	
Ex‐smoker	10 (31.3%)	21 (30.9%)	31 (31.0%)	
Never smoked	13 (40.6%)	27 (39.7%)	40 (40.0%)	
Alcohol abuse				0.621
Yes	7 (21.9%)	18 (26.5%)	25 (25.0%)	
No	25 (78.1%)	50 (73.5%)	75 (75.0%)	
Previous radiotherapy				0.748
Yes	5 (15.6%)	9 (13.2%)	14 (14.0%)	
No	27 (84.4%)	59 (86.8%)	86 (86.0%)	
Poor dentition				0.259
Yes	8 (28.6%)	10 (17.9%)	18 (21.4%)	
No	20 (71.4%)	46 (82.1%)	66 (78.6%)	
ASA				0.413
1 or 2	15 (46.9%)	26 (38.2%)	41 (41.0%)	
3 or 4	17 (53.1%)	42 (61.8%)	59 (59.0%)	
Diabetes mellitus				0.096
Yes	11 (34.4%)	13 (19.1%)	24 (24.0%)	
No	21 (65.6%)	55 (80.9%)	76 (76.0%)	
Preoperative hypoalbuminemia				0.258
Yes	4 (12.9%)	3 (5.77%)	7 (8.43%)	
No	27 (87.1%)	49 (94.2%)	76 (91.6%)	
Preoperative anaemia				**0.002**
Yes	14 (43.8%)	10 (14.9%)	24 (24.2%)	
No	18 (56.3%)	57 (85.1%)	75 (75.8%)	
Tracheostomy				0.670
Yes	23 (71.9%)	46 (67.6%)	69 (69.0%)	
No	9 (28.1%)	22 (32.4%)	31 (31.0%)	
Neck dissection				0.348
Yes	26 (81.3%)	60 (88.2%)	86 (86.0%)	
No	6 (18.8%)	8 (11.8%)	14 (14.0%)	
Wound class				0.802
Clean	8 (25.0%)	19 (27.9%)	27 (27.0%)	
Clean‐contaminated	24 (75.0%)	49 (72.1%)	73 (73.0%)	
Flap type				**0.030**
ALT or RF	20 (62.5%)	56 (82.4%)	76 (76.0%)	
Other	12 (37.5%)	12 (17.6%)	24 (24.0%)	
Tissue type				0.391
Osseous component	8 (25.0%)	12 (17.6%)	20 (20.0%)	
Soft only	24 (75.0%)	56 (82.4%)	80 (80.0%)	
Hardware use				0.190
Yes	11 (34.4%)	15 (22.1%)	26 (26.0%)	
No	21 (65.6%)	53 (77.9%)	74 (64.0%)	
T‐stage				0.497
1 or 2	10 (31.3%)	26 (38.2%)	36 (36.0%)	
3 or 4	22 (68.8%)	42 (61.8%)	64 (64.0%)	
Reason for operation				0.330
Cancer	29 (90.6%)	65 (95.6%)	94 (94.0%)	
Other	3 (9.40%)	3 (4.40%)	6 (6.00%)	
Antibiotic prophylaxis choice				0.159
Cephazolin	3 (19.4%)	17 (25.0%)	20 (20.0%)	
Cephazolin + metronidazole	15 (46.9%)	20 (29.4%)	35 (35.0%)	
Cephazolin + metronidazole + amoxicillin/clavulanic acid	5 (15.6%)	15 (22.1%)	20 (20.0%)	
Other	9 (28.1%)	16 (23.5%)	25 (25.0%)	
Antibiotic prophylaxis duration				**0.013**
≤ 24 h	13 (40.6%)	12 (17.6%)	25 (25.0%)	
> 24 h	19 (59.4%)	56 (82.4%)	75 (75.0%)	
Antibiotic prophylaxis duration				0.311
≤ 48 h	19 (59.4%)	33 (48.5%)	52 (52.0%)	
> 48 h	13 (40.6%)	35 (51.5%)	48 (48.0%)	
Antibiotic prophylaxis duration				0.515
≤ 72 h	21 (65.6%)	40 (58.8%)	61 (61.0%)	
> 72 h	11 (34.4%)	28 (41.2%)	39 (39.0%)	
Incision through mucosal surface				0.670
Yes	46 (67.6%)	23 (71.9%)	69 (69.0%)	
No	22 (32.4%)	9 (28.1%)	31 (31.0%)	

Multivariate analysis				
Variable	*p*	Odds ratio	95% confidence interval	
			Lower	Upper
Duration (≤ 24 vs. > 24 h)	**0.041**	3.010	1.045	8.674
Preoperative anaemia (yes vs. no)	**0.025**	3.259	1.159	9.169
Flap type (other vs. ALT or RF)	**0.032**	3.157	1.100	9.059

*Note: p* < 0.05 is statistically significant.

^a^ Mann–Whitney *U* test used due to a significant Levene's test (*p* = 0.036).

^b^ Healthy weight: 18.50–24.99 kg/m^2^.

^c^ Unhealthy weight: < 18.50 or ≥ 25.00 kg/m^2^.

The antibiotic regimens used for these cases varied, with 16 different combinations in total. A cefazolin and metronidazole combination was the most common regimen for patients in both the ≤ 24 and > 24 h prophylactic antibiotic groups (40.0% and 32.0%, respectively). Cefazolin monotherapy was also quite common for both groups (24.0% and 18.7%, respectively). The mean duration of antibiotic prophylaxis was 3.82 days, with 25% receiving prophylactic antibiotics for less than or equal to 24 h post‐surgery and 75% receiving greater than 24 h of prophylaxis.

## 4 Discussion

There have been several studies which have examined the rate of SSI following microvascular free flap reconstruction of the head and neck. Reported infection rates range greatly from as little as less than 10% to greater than 40% [5, 7, 8, 9, 10, 11, 12, 13, 14]. The 32.0% infection rate reported in this study sits towards the upper end of this range and is noticeably greater than the 20.3% recipient SSI rate reported in a comparable Australian study by Gearing et al. at The Royal Melbourne Hospital [12].

Our analyses showed SSI to be significantly associated with longer length of stay as well as higher rates of unplanned return to theatre and complete flap failure. Lindeborg et al. analysed 282 cases of head and neck free flap reconstructions and found SSI to significantly increase length of stay [20]. Similarly, Goyal et al. found this to be the case in pedicled flap reconstruction in the region [21]. The 14‐day median length of stay observed in our study is greater than the 10‐day median reported by Lindeborg et al. and the 9.3‐day mean found by Goyal et al. [20, 21] The increased length of stay in our study could be attributed to a substantially higher SSI rate, with the other studies reporting rates of 8.9% and 9.1%, respectively [20, 21]. Zhao et al. observed SSI in free flap reconstruction of the head and neck to be an independently significant post‐operative predictor of unplanned reoperation [4]. Our study did not capture the timing of return to theatre, and therefore a causative relationship with SSI cannot be determined from our results. Furthermore, SSI has been associated with complete flap loss by Mücke et al., with similar results being reported in other studies [21, 22, 23].

To the author's knowledge, this is the first paper to establish preoperative anaemia as an independently significant risk factor for SSI in head and neck free flap operations (OR: 3.259). A plausible mechanism is reduced haemoglobin‐mediated oxygen delivery to peripheral tissues. Adequate oxygenation is critical for cellular proliferation, angiogenesis and protein synthesis, as well as the oxidative bactericidal mechanism of leukocytes [24, 25, 26]. Taken together, these mechanisms may explain the observed association between anaemia and increased risk of SSI in our cohort. Ramos‐Zayas et al. found presurgical anaemia to be a significant risk factor for hospital‐acquired infections following microvascular head and neck reconstruction [9]. Unlike their study, ours focused exclusively on SSIs, which represent only one component of the broader category of hospital‐acquired infections examined in their work. An analysis of 113 891 patients by Campbell et al. aimed at defining best practices to prevent SSI found hospitals with low SSI rates were less likely to have patients presenting to the operating theatre anaemic [27]. This study, however, examined SSIs in general, rather than those specifically following head and neck free flap reconstructions. For these operations, other papers have associated surgical blood loss and perioperative blood transfusion with SSI, but none have made an association with preoperative anaemia [10, 13, 14, 28, 29]. Considering that head and neck reconstructions are typically semi‐elective procedures, presurgical intervention is possible to reduce the likelihood that patients are anaemic come the day of surgery.

Flap type has been a relevant risk factor for SSI in several papers studying head and neck reconstruction. Mücke et al. analysed 156 bony flap reconstructions of the head and neck and found iliac crest flaps to result in significantly more SSIs than free fibular flaps [22]. Various studies have also observed bony flaps to significantly increase SSI rates compared to free flaps that were composed of only soft tissue [12, 30, 31]. It should be noted that our study did not find a significant difference in SSI rate between osseous and soft‐tissue‐only flaps (*p* = 0.391). Our study did, however, find the use of flaps other than ALT or RF free flaps to be an independently significant risk factor for SSI (OR: 3.157). This result may reflect the increased complexity and risk factors associated with the other flap types. The most common flap type in the ‘other’ category was the free fibular flap (*n* = 18). These reconstructions also have a longer mean operative duration (12.6 vs. 11.3 h) and a greater rate of hardware use (87.5% vs. 6.6%). The reliable perfusion and soft‐tissue bulk of the ALT and RF may confer some measure of protection against infection; however, this is a theoretical benefit that would be difficult to prove.

The optimal duration of antibiotic prophylaxis in free flap reconstruction of the head and neck is contentious. Conflicting results have been reported by recent systematic reviews and meta‐analyses [32, 33, 34, 35]. Haidar et al. found that ≤ 24 h of appropriately chosen antibiotic prophylaxis is likely sufficient; however, the authors agree that a strong conclusion remains elusive [32]. In contrast, a more recent systematic review and meta‐analysis by Vander Poorten et al. found that a duration of prophylaxis of 24–48 h prevented SSIs most effectively in clean‐contaminated head and neck surgery [34]. When reviewing these meta‐analyses, however, it is worth highlighting that many of the included studies employed varying definitions for SSIs, and the patient populations varied greatly, making comparisons difficult. Our study adds to the body of evidence by identifying that an antibiotic prophylaxis duration of ≤ 24 h significantly increased SSI rates by three times compared to prolonged courses for head and neck free flap reconstruction. A similar result was found in the Australian study by Gearing et al., who determined that short courses (≤ 24 h) of prophylactic antibiotics significantly increased SSI rates by 2.33 times [12].

Current Australian guidelines recommend that antibiotic prophylaxis should not extend beyond 24 h in head and neck operations [15]. Assuming that the optimal length of prophylactic antibiotics is the shortest period of antibiotic use with non‐inferior infection rates, our results suggest that 48 h may be superior for free flap reconstructions, with no significant difference in infection rate at this cut‐off point.

Several other factors have been shown to increase the risk of SSI in the head and neck population. Patient characteristics such as sex, diabetes, hypoalbuminemia and history of alcohol abuse have all been found as predisposing features for SSI in head and neck free flap reconstruction [12, 13, 27, 31, 36]. Operative duration and tracheostomy have been found to contribute to SSI [9, 12, 36], while flap characteristics, such as size, donor site and tissue type, also appear to play an important role [6, 12, 30]. In addition, the T‐classification of the resected tumour before flap reconstruction has been demonstrated to contribute to SSI [10, 12]. Despite the results in the studies cited above, all these factors were found to not be significant in our study as well as in other relevant investigations.

## Author Contributions

**Marco Stocca:** data curation, formal analysis, writing – original draft. **Pascalino Romeo:** formal analysis, methodology, writing – original draft. **Yasiru Gehan Karunaratne:** formal analysis, methodology. **Bishoy Soliman:** formal analysis. **Thomas C. Lam:** resources, supervision. **Huang‐Kai Kao:** methodology, supervision. **Frank Hsieh:** conceptualization, formal analysis, project administration, supervision, writing – review and editing.

## Disclosure

The authors have nothing to report.

## Conflicts of Interest

The authors declare no conflicts of interest.

## Data Availability

The authors have nothing to report.
